# Comments on the Action of the American Society of Dental Surgeons

**Published:** 1848-04

**Authors:** B. B. Brown

**Affiliations:** St. L. Ed.


### COMMENTS,

### *On the action of the American Society of Dental Surgeons.*

BY B. B. BROWN, M. D. D. S.—ST. LOUIS ED.

We had hoped that we might succeed in avoiding any *controversial* participation in the subject of "Amalgams," but a solemn duty demands that we should lay before our readers a few facts, connected with ourself, in relation to this vexed question. And while we disclaim that we have any personal motives to gratify, or any private hostility to indulge, yet we shall ever pay due deference to those high objects, which involve, in their consequences, the fundamental principles upon which the profession is based. It is a matter of the deepest importance to every member, to preserve the character of the profession, as well from the tyranny of the *few*, as the charlatanism and quackery of the many. We shall ever vindicate it from the absurd idea, that a Dental Surgeon cannot practise, with honor, unless it be under the sanction and obligation of a *written* PLEDGE, which shall be made conformable to the freaks and caprices of a *self-elected*, and *self-constituted* junto, desirous of arrogating to itself the dignity of the Reformer. A modern Cervantes might find admirable matter for ridicule in the *profoundly comprehensive* operations of these Don Quixotes! We feel a deep and ardent interest in the progress of our profession, and believe that, in point of intelligence and worth, it is not behind any other profession of the present times; and that individual members are fully qualified to act and to think for themselves, in the prosecution of their practice, and the management of their professional business, without requiring the interposition of the Court in Chancery, to settle the question *non compos mentis*, and to place the unfortunate parties under strict and vigilant guardianship.

The "American Society of Dental Surgeons," at its last annual meeting, adopted resolutions which are hostile to the harmony, and to the spirit and purposes which originated that Institution. No member of a liberal and learned profession can view such principles in any other light, than as a gross and premeditated outrage, if carried by a majority of the members, upon the rights, the privileges, and the characters of the minority. But consummated, as it was, by a very small *minority* of ALL the members of the Society, the act, we venture to aver, finds no parallel in the annals of the Inquisition, or in the Draco—code of tyrants. To show the purely *malignant* and tyrannical character of the whole procedure, we shall instance our own case. We do not now, and have not for many years, used any "*amalgams*," and *then only for experiment*, as the correspondence plainly shows—that we have fulfilled every moral and professional obligation towards our associates, and that we have ever labored to advance the interests of the profession, and the character of its members. And yet, we have incurred the animosity of that *sage* minority, who unfortunately held the destinies of the Society, for the time being, in their very able and distinguished keeping. We *refused* to PLEDGE ourself! We refused to acknowledge the right of our associates to introduce a *new* test of membership, and to apply it, in the most *offensive* and *indelicate* manner, to ascertain the *fitness* and *capacity* of their compeers—a Procrastean bed, on which the *short* were quacks, and the *long* were empyrics. We *refused* to sign a "PLEDGE" to do what we *had* not done, and what we did not *intend* to do. We *refused*, in order to vindicate our own dignity, as well as the dignity of the profession, and to preserve our self respect, as one of the members of a learned association.

The Resolutions and Protest were published in the second number of the Register, see page 95, 96, and to which the following letter is in reply.

*St. Louis, October 20th*, 1845.

MY DEAR SIR:

The printed Resolutions, and blank Protest, of the "American Society of Dental Surgeons," were received in due time. In reply, I have only to say, that my professional attainments and character occupy higher ground, than to be compelled to forfeit my self respect, by submitting to the requirements of resolutions, whose *tone* is *threatning*. The Society has transcended its powers, has violated the compact which ushered it into existence, by enacting resolutions which are arbitrary, unjust, and unconstitutional, and, I believe, unless they are abandoned, the fate of the Society is sealed, or I have mistaken the signs of the times.

I do not use any amalgam, or cement, whatever; and have invariably, when consulted by my professional brethren in the West, recommended the following substances, or Foils, for filling teeth, viz: Gold and Platinum;—Gold, however, has advantages over all other substances. Tin and Lead are admissible; Tin possesses advantages over Lead, and the demand for the latter is less frequent. In my own practice, Gold has always been preferred, and most extensively used; Platinum, Tin and Lead have also been used, but only to a limited extent, particularly the latter.

When the Society recommended the profession to abandon the use of Amalgams and cements for the purpose of filling teeth, it did not travel out of the bounds of its duty,—the recommendation was right and proper, and every honorable and high minded practitioner of Dental Surgery, whether a member of the Society or not, I venture to aver, was to be found on the side of the Society, and opposed to the indiscriminate and general use of amalgams, cements, &c., &c.; but, when the Society declare the use of these objectionable materials to be *empyricism*, it goes one step too far. I saw that declaration with much regret, and, when I read the Resolutions and Protest of the last meeting, I confess it was with feelings of pain and mortification.

In conclusion, I will say that my membership of the Society does not, I humbly trust, rest upon such a doubtful foundation, as to subject me to the liability of expulsion, unless I sign a *certificate*, and make a *pledge*. When a member is supposed to have violated the Constitution or By-laws of the Society, he should be arraigned under their provisions, and, if found guilty, expelled.

I will now rest the matter for the present, and await the issue.

Your Ob't Serv't,

AMOS WESTCOTT, M. D. D. D. S., B. B. BROWN.

*Rec. Sec'y, A. S. D. S.*

P. S. Will you have the kindness to inform me, (at your convenience,) what has been done in relation to the matter at issue?

B. B. B.

The following is Dr. Westcott's reply.

*Syracuse, October 30th,* 1845.

MY DEAR SIR:

Yours of the 20th inst., is this moment received and read, and in answer to your request, I reply, that if the

American Society of Dental Surgeons have no authority to proscribe quackery among its members, it has *no object*, and the sooner it ceases to exist, the better. *Yours* is surely a new objection, save in a single instance, and one in my judgment, as having not the slightest foundation.

You ask me to inform you "what has been done in relation to the matter at issue." If I apprehend the import of your interrogation, I can only reply, that the Society have passed resolutions upon this subject, clearly pointing out a course, with a view to suppress this *worst kind* of *quackery*—these resolutions have been forwarded to every member, accompanied with a pledge in accordance therewith—the Society appointed me as their agent, to carry out its views, and made my duty definite and explicit. I shall therefore, unless I receive new instructions, (and these must come through a special session,) carry out *most strictly* the *original order*—which order you have in the circular I sent you.

Very respectfully yours,

A. WESTCOTT.

The following letter is in reply to one on the same subject, from my friend, Professor Harris;—inasmuch as the correspondence was not official, I cannot take the liberty of publishing the letter without his consent.

*St. Louis, July 14th*, 1846.

My Dear Doctor:

Relative to the use of "amalgams," I have only to say, that I addressed a letter on the subject to Dr. Westcott, which was published (not entire) in the Journal,—page 170, vol. VI. My opinion has undergone no change in relation to the matter, since that period.

The temperate and elegant letter of Dr. C. C. Allen, which was published in the Journal, vol. VI., beginning at page 246, embodies my own views and sentiments as fully, and probably more so, than I could hope to express them. The belligerent reviews,

(to say nothing of the sophistry which they contain,) published in the Journal from time to time, relative to amalgams—protest of the Society, &c., I conceive are uncalled for, to sustain a tenable position, nor are they calculated to advance the interest and dignity of the Dental profession.

With the highest regard,
I have the honor to be
Your ob't servant,
B. B. BROWN.

PROF. C. A. HARRIS.

I received another Protest, dated December 15th, 1846, equally as objectionable as the former, and like it in every respect, except this omission, "not only unfit, but dangerous," and to which the following letter is a reply.

*St. Louis, July 13th,* 1847.

MY DEAR DOCTOR:

I have received another certificate, requiring my signature in relation to Amalgams, &c., &c., I regret that this second form is even more objectionable than the first, rendered so, I must confess, by the obstinate repetition of the subject. Every formula, which requires the members of a learned profession to be *pledged*, like lost drunkards at a temperance meeting, is, in my humble opinion, an outrage upon the profession itself, and revolting, in the highest degree, to the personal dignity of the gentleman and the scholar. In the nature of things, each member of this learned body must practise in accordance to the dogmas of science, as received by the learned, at the same time, however, exercising his best judgment in reference to the adoption, or non-adoption, of any remedial agent which research, together with careful experiment, may have placed within his reach.

All attempts to interfere, in this objectionable manner, with the *catholic spirit* which animates the profession, must terminate in arrant quackery, and force the society into the unenviable position of being the laughing stock of the more intelligent and respectable portion of the community. I am ever disposed to respect the opinions of those who differ with me, when I am satisfied that those opinions are the result of a conscientious and scrupulous examination of all the facts which go to govern the case. The wisest head often draws wrong conclusions from the best of premises,—the ablest manipulator makes, frequently, unfortunate mistakes, and the most cautious experimentalist, is led at times into irretrievable difficulties; but, my dear friend, in all this *there is no quackery whatever.*

The dark cloud of error can alone be dispelled by able and gentlemanly discussion, carried on in a spirit of candor and forbearance towards every member of the profession. The day has long since passed by, in which the *ipse dixit* of any man, or even of a dozen men, could authoritatively, and *ex cathedra*, stamp this opinion as heterodox, or that practice as quackery. I repeat, that I do not use amalgams of any kind in my practice. Gold and tin are the only substances used by me for filling teeth. I prefer gold, and hence, urge its use in all cases where the patient can incur the expense.

Pardon my egotism, my friend, when I say that, during the last fifteen years, I have used every exertion to be, if possible, in advance of my profession; and when the literature which belonged to it did not seem to enlighten me sufficiently, I have had recourse to the cognate sciences, for further information. Science is open to the investigations of all men, and he who will not apply some of the results of his investigations to practice, is at least but a poor student, and must always remain a very indifferent practitioner.

I deprecate the use of amalgams in dental practice, having long since tested their claims to usefulness, and their character, by experiment and analysis. I hope and trust that you will, my dear friend, move to lay this whole matter, in relation to amalgams, upon the table, thereby rendering the Dental profession a lasting service, and adding another leaf to the chaplet of your well deserved fame. For this amalgam war, as it is now waged, can end in no useful purpose to the profession; indeed, it has in some of its details, already assumed the disgraceful characteristic of newspaper ribaldry, and personal recrimination.

The Society may take such action as shall please it best in reference to myself. Even if it had the power to require *pledges* of me, as a man I am bound, absolutely and without qualification, to refuse to give them. But I deny the constitutionality of the Society's action in the premises, and shall not quietly submit my standing, as a Dental Surgeon, to be compromised by the unconstitutional efforts of my associates.

I have to request that you will act as my proxy at the approaching annual meeting of our Society, if I should fail to be present, and that you will make such use of this letter as you may think proper. With the highest regard,

I have the honor to be

Your ob't servant,

B. B. BROWN.

P. S. As I have not replied to Dr. Westcott, will you have the kindness to make an apology to him for this seeming dereliction of duty. I thought it best that my letter should be in the hands of my proxy. B. B. B.

PROF. C. A. HARRIS.

At the annual meeting of the American Society of Dental Surgeons, held August 3d, 1847, the Recording Secretary reported the names of twenty-one gentlemen as delinquents. who refused to sign the pledge, to wit,—C. C. Allen, E. Baker, Geo. E. Haws, John Lovejoy, E. Laroque, D. Morrow, Geo. Merriman, C. S. Rowell, E. M. Bissel, S. Dillingham, J. Smith Dodge, Nehemiah Dodge, P. Houston, E. Jamet, Benj. Lord, Jas. Taylor, Geo. W. Parmley, Elijah Bryan, N. C. Keeps, I. M. Peak and B. B. Brown. In the progress of business their cases were taken up, and seperately disposed of. Some sacrificed their principles, as previously expressed by themselves, and, we doubt not, their self respect, by succumbing to the imperious action of that small and factious majority then present—but vastly in the minority as it regards the whole society. Some others, who were not present, by the efforts of friends had the matter laid over until the next annual meeting; when, we trust, a determined opposition will be able to restore the harmony of the Association, and preserve it from the worse than Vandalism, which, at its recent meeting, characterized its proceedings. We cannot, however, leave this subject, without tendering our sincere respects and hearty gratitude, to those few brave spirits, who disputed every inch of ground, opposed with vigor the introduction and adoption of so unprofessional a proceedure; and those of them who fell, fell as brave men ever fall, nobly contending for right and justice, as regards their associates, and the best interests of the "American Society of Dental Surgeons."

While we differ with those who advocate the use of "amalgams," for filling teeth, we cannot, however, avoid admiring the forbearance and high tone of those who opposed the action of the Society. As we were among those who fell, without an opportunity of vindicating ourself in person, "Bowie knifed" and "Broadaxed," for aught we know to the contrary, yet circumstances of a *peculiar character* have prevented our total annihilation, and we are allowed, by a good Providence, still to survive the shock, and hope to live long enough to see the outraged honor of the Dental profession, fully vindicated.—ST. L. ED.

